# The effects of positive versus negative impact reflection on change in job performance and work-life conflict

**DOI:** 10.3389/fpsyg.2014.01370

**Published:** 2014-11-27

**Authors:** M. Teresa Cardador

**Affiliations:** School of Labor and Employment Relations, University of Illinois at Urbana-ChampaignChampaign, IL, USA

**Keywords:** task significance, impact reflection, perceived impact, positive impact, negative impact

## Abstract

Research on task significance and relational job design suggests that information from beneficiaries of one’s work fosters perceptions of impact, and thus improved work outcomes. This paper presents results from a longitudinal field experiment examining the effect of another strategy for fostering perceptions of impact – engaging employees in regular reflection about how their work benefits others. With a sample of professionals from multiple organizations, this longitudinal study examined the effect on job performance and work-life conflict of both positive and negative impact reflection. Results show that negative impact reflection had a pronounced negative effect on job performance, but no effect on work-life conflict. Positive impact reflection had a weak positive effect on work-life conflict, but no significant effect on job performance. The direction of effects seen in the no intervention condition mirrored that of the negative impact reflection condition, suggesting a possible buffering effect for positive impact reflection. This research provides empirical and theoretical contributions to the literatures on relational job design and task significance.

## INTRODUCTION

Employees are increasingly interested in doing work that makes a difference (Colby et al., 2001), and organizations – recognizing the benefits – are actively looking to provide employees with opportunities to experience their work as more impactful (Grant, 2011). In line with this interest, job design scholars have recently given renewed attention to strategies that might further employees’ sense of impact at work (Grant, 2007). The purpose of this research is to test the effect of one such strategy – impact reflection.

Traditional job design research suggests that task significance – judgments that one’s work has a positive impact on others – stem from the objective features of tasks (Hackman and Oldham, 1976). This research takes the view that some jobs naturally afford more opportunities for task significance, and that impact perceptions can be fostered by structurally enriching the task characteristics of workers. More recently, however, job design researchers have adopted a social information processing perspective on task significance (Salancik and Pfeffer, 1978; Zalesny and Ford, 1990; Pollack et al., 2000; Parker et al., 2001; Morgeson and Campion, 2003), suggesting that impact perceptions are subjective and shaped by the salience of the social context surrounding one’s tasks (Grant, 2008a). Studies of this approach – termed relational job design – have shown that impact perceptions are increased when employees have interpersonal contact with, or receive social information from, beneficiaries of their work (e.g., Grant, 2007, 2008b; Grant et al., 2007; Grant and Hofmann, 2011).

While recent studies adopting a social information processing perspective on task significance have provided important insights, they are subject at least two limitations addressed through the current research. First, experimental research has focused primarily on the effect of interventions that help workers process information that comes *from* the beneficiaries of their work (Grant et al., 2007). However, given that it is not always practical to redesign jobs to provide contact with or information from beneficiaries (Northcraft and Chase, 1985), it may be important to consider other strategies. The fact that individuals may also process social information *about* existing social interactions (Festinger, 1954; Salancik and Pfeffer, 1978) raises the possibility that engaging workers in reflection about impact may also affect task significance perceptions, and work outcomes. Reflection strengthens and lengthens the intensity of recollection of the event or action that is being recalled (Usher and Bryant, 1989). Accordingly, events should become more salient when individuals reflect on them. Indeed, there is already some evidence that engaging in reflection about the impact of one’s work may have an influence on workers. In both laboratory and field settings with undergraduate students, Grant and Dutton (2012) showed that encouraging individuals to reflect on the experience of helping others (versus being helped by others) improved their prosocial behavior 2 weeks later. However, the effect of impact reflection has not yet been tested in a professional field setting.

Second, as noted by Grant (2008a) and Grant and Dutton (2012), previous research has primarily focused on outcomes associated with enhancing perceptions of *positive* impact without examining the potential effects of *negative* impact perceptions – judgments that work has a negative impact on others (Grant and Campbell, 2007). Because many jobs necessitate doing things that have a negative impact on others [e.g., managers firing employees or giving negative feedback (Molinsky and Margolis, 2005)], workers can perceive jobs as having both positive and negative impact (Grant and Campbell, 2007). Thus, it may be important to also consider the effect of cues that trigger perceptions of negative impact at work.

The purpose of this study is to address these empirical and theoretical limitations of the literature on relational job design and task significance by testing the effect of positive and negative impact reflection on two outcomes previously examined in the relational job design literature – job performance and work-life conflict. While previous research on relational job design has examined the relationship to more “proximal” outcomes such as social worth and prosocial behavior (Fried and Ferris, 1987; Grant, 2008a), research has also demonstrated the effect of positive impact cues on changes to more “distal” outcomes, such as job performance and work-life conflict (Grant, 2008a; Sonnentag and Grant, 2012). This latter evidence suggests that the two month time frame in this study should be sufficient to capture change in these more distal outcomes.

Because reflection is an important part of processing social information about work (Morris, 1989), I expect that engaging in impact reflection should have an effect on workers, and on work outcomes. In particular, based on prior research, I expect that positive impact reflection will increase job performance and decrease work-life conflict. Conversely, I expect that negative impact reflection will decrease job performance and increase work-life conflict. Job performance refers to behaviors that support or contribute to task effectiveness (Motowidlo et al., 1997). Work-life conflict is a form of inter-role conflict in which pressures from the work and home/family are incompatible in some respect (Kopelman et al., 1983). Though role incompatibility can be bi-directional (Greenhaus and Beutell, 1985), my focus here is on work to home/family conflict.

### OVERVIEW OF THE CURRENT STUDY

Building from existing research, I apply the protocol used by Emmons and McCullough (2003), and adapted by Grant and Dutton (2012), to the study of impact reflection and its effects on employee experiences at, and outside of, work. I conducted a longitudinal field experiment with professionals in multiple organizational contexts, in which I engaged workers in weekly reflection (over an 8 week period) about the positive or negative impact of their work on others. This study allows for an examination of whether individual job performance and work-life conflict can be influenced through an intervention designed to encourage repeated reflection about how one’s work makes a positive or negative impact.

## MATERIALS AND METHODS

### PARTICIPANTS AND PROCEDURE

Participants were recruited from alumni of a master’s degree program in human resource management and industrial relations in the US. Alumni were recruited through an email solicitation that briefly described participation requirements, and indicated that eligible participants must work at least 30 h per week. The email also described that participants would be given a $25 Amazon gift card for their involvement. Two hundred and twenty (220) people replied that they were interested in participating in the survey. Using a random number generator, these individuals were randomly assigned to the positive impact reflection condition, the negative impact reflection condition, or the control group. Of the 220 respondents who expressed interested in participating in the study, 217 individuals completed the Time 1 (pre-intervention) survey – *N* = 73 in the positive impact reflection condition, *N* = 74 in the negative impact reflection condition, and *N* = 70 in the control group. Of the 217 who completed the Time 1 (pre-intervention) survey, 176 (81.1%) completed the Time 2 (post-intervention) survey 9 weeks later.

The sample mean age was 36.5 (SD = 10.8). The sample was 65% female at Time 1 and 68% female at Time 2. Attrition analyses showed no significant pre- and post-intervention differences in age, *t*(214) = 1.04, *p* = 0.30; however, females were more likely to remain in the study, *t*(215) = -2.06, *p* = 0.04. No information was collected on participant ethnicity. To further examine the differential attrition rate between men and women, I examined gender attrition rates for each condition separately. I found no significant gender differences in attrition by condition^1^.

The 217 participants represented 130 unique organizations [78.5% were for-profit organizations (Fortune 500 or 100 corporations), and 20.4% were non-profit organizations (higher education, government, or other non-profit organizations)]. For 1.1% of the sample, organization type could not be determined because organizational information was not provided. Attrition analysis revealed no significant pre- and post-intervention sample differences in organization type, *t*(213) = 1.97, *p* = 0.16.

At Time 1 (pre-intervention), all participants completed an online survey comprised of the measures of job performance and work-life conflict. Before beginning the Time 1 survey, participants read and electronically provided informed consent. Consistent with the language approved by the sponsoring University’s Institutional Review Board, the consent form described the study, and provided information about participant rights and risks, as well as who to contact with questions or concerns about the study. Approximately 1 week later, participants completed the first weekly impact reflection exercise. Participants were contacted via email on the same day, and approximately the same time each week, for a total of 8 weeks. They were provided with an online survey link to that week’s impact reflection exercise.

The reflection exercise, adapted from the protocol used by Emmons and McCullough (2003), was designed to take no more than 5 min to complete. In the Emmons and McCullough’s (2003) protocol, individuals were randomly assigned to either a grateful outlook or daily hassles condition. Individuals in the grateful outlook condition engaged in weekly reflection about things for which they were grateful, while those in the daily hassles condition engaged in weekly reflection about struggles and inconveniences they had experienced. Adapting this paradigm, participants received positive or negative impact prompts. In the positive impact reflection condition participants received the following weekly prompt:

There are many ways, both large and small, that our work may have a positive impact on others. Think back over this work week and record in the space below five (5) ways that you had a positive impact on others through your work/job.

Consistent with established methods (Emmons and McCullough, 2003), participants in the negative impact reflection condition were prompted instead:

There are many ways, both large and small, that our work may have a negative impact on others. Think back over this work week and record in the space below five (5) ways that you had a negative impact on others through your work/job.

Instead of engaging in weekly reflection about their impact, participants in the control condition answered a brief questionnaire – designed to take the same amount of time as the reflection exercise – about what they did that week. Participants were prompted with the following:

Think back over the last week at work and indicate whether you did the following activities.

The 10 activities listed were intended to be “neutral” and included: “Cleaned my desk” and “Bought a cup of coffee on my way to work.” Participants answered “yes” or “no” as to whether they did each of the ten activities in the last week. To ensure that the control condition remained neutral, I did not provide participants with opportunities for open-ended reflection.

Participants in the positive impact reflection condition submitted a total of 2,484 reflections (an average of 4.5 per person per week), and participants in the negative impact reflection condition submitted a total of 1,241 reflections (an average of 3.3 per person per week). This difference in number of reflections provided may suggest a difference in participants’ ease of recall of instances of negative versus positive impact.

One week following completion of the final weekly impact reflection exercise, participants completed the Time 2 (post-intervention) survey consisting of the same measures of job performance and work-life conflict, as well as the positive and negative impact manipulation checks. After study completion, participants were electronically given a study debrief informing them about the method and intent of the study.

### MEASURES

For all measures, respondents indicated the extent to which they agreed with each item (1 = *Strongly Disagree* and 5 = *Strongly Agree*).

#### Job performance

Participants responded to a 5-item job performance scale (Williams and Anderson, 1991). Items included: “I adequately complete assigned duties” and “I meet performance requirements of the job” (α = 0.91 at Time 1, 0.90 at Time 2).

#### Work-life conflict

Participants completed a 6-item work-life conflict scale adapted from Kopelman et al. (1983). Items included: “When I get home from work I am often too frazzled to participate in home activities” (α = 0.90 at Time 1, 0.91 at Time 2).

#### Manipulation checks

To check that the intervention was producing the desired effect, participant perceptions of positive and negative impact were measured post-intervention. Positive impact was assessed using a single item from Grant (2008a): “I feel that my work makes a positive difference in other people’s lives.” Negative impact was measured using a single item from Grant and Campbell (2007): “My work can often negatively impact others.” For an additional check of the manipulation, I examined participant impact reflections to ensure that entries were consistent with the positive or negative impact prompt. The author and a graduate student blind to the study’s purpose, coded all entered reflections as “positive impact,” “negative impact,” or “unable to tell.” Both raters were able to categorize all but five reflections into either the “positive” (2,480 reflections) or “negative” (1,240 reflections) categories. Five reflections were coded by both raters as falling into the “unable to tell” category. Accordingly, the two raters achieved a 100% inter-rater match.

To test for condition-specific attrition, I compared Time 1 and Time 2 participation across the three conditions. I found significant differences in attrition between the negative impact reflection condition and both the positive impact reflection and control conditions, *F*(2,216) = 7.40, *p* = 0.001. Sixty-eight percent (68%) of those in the negative impact reflection condition completed the Time 2 survey, compared with 89% in the positive impact condition, and 86% in the control condition.

## RESULTS

Means and SDs, and bivariate correlations for all variables appear in **Table 1**.

**Table 1:** Means, SD, and bivariate correlations for all variables.

Variable	Mean (SD)	1	2	3	4	5	6	7
1. Condition	2.00 (0.82)	-						
2. Average number of reflections	3.93 (1.46)	0.48**	-					
3. Job performance *(Time 1)*	4.61 (0.45)	-0.20	0.12	-				
4. Job performance *(Time 2)*	4.48 (0.48)	0.14	0.19*	0.52**	-			
5. Work-life conflict *(Time 1)*	2.92 (0.96)	-0.04	-0.03	0.02	0.01	-		
6. Work-life conflict *(Time 2)*	2.90 (1.02)	0.02	0.02	0.05	0.01	0.77**	-	
7. Positive impact *(Time 2)*	3.62 (0.74)	0.14	-0.02	0.03	0.13	-0.11	-0.19*	-
8. Negative impact *(Time 2)*	1.94 (0.86)	-0.11	0.01	-0.11	-0.06	0.05	0.14	-0.06

Time 1 N = 217, Time 2 *N* = 176; **p < 0.01, *p < 0.05.

### MANIPULATION CHECKS

It was expected that the effect of the impact manipulation would be higher levels of perceived positive impact in the positive impact reflection condition (as compared to the negative impact reflection and control conditions), and higher levels of perceived negative impact in the negative impact reflection condition (as compared to the positive impact reflection and control conditions). Analysis of variance (ANOVA) revealed significant differences in perceived positive impact across conditions, *F*(2,175) = 2.97, *p* = 0.05, η^2^ = 0.03 (positive impact: μ = 3.8, SD = 0.62; negative impact: μ = 3.5, SD = 0.78; control: μ = 3.5, SD = 0.81). Contrast analysis showed that reported levels of perceived positive impact were significantly higher in the positive impact reflection condition as compared to the other two conditions, *F*(10,175) = 5.84, *p* = 0.02, η^2^ = 0.03. Similarly, ANOVA showed significant differences in perceived negative impact across conditions, *F*(2,175) = 5.83, *p* = 0.01, η^2^ = 0.04 (negative impact: μ = 2.2, SD = 0.99; positive impact: μ = 1.9, SD = 0.84; control: μ = 1.8, SD = 0.70). Contrast analysis indicated that reported levels of perceived negative impact were higher in the negative impact reflection condition as compared to the other two conditions, *F*(1,175) = 5.75, *p* = 0.02, η^2^= 0.03.

As noted, participant reflection entries were content analyzed as an additional check of the manipulation. As expected, participants in the positive impact reflection condition entered reflections appropriate to positive impact, such as: “Helped a team member with especially difficult cases,” and “Coached a direct report to help them develop.” Participants in the negative reflection condition entered reflections consistent with the construct of negative impact, such as: “Let an employee go for attendance issues,” and “I had to provide negative feedback to a supervisor about their performance.” Together, these results confirm the effectiveness of the impact manipulations.

Means, SDs, and mean changes for job performance and work-life conflict appear in **Table 2**. To assess the effects of the intervention, I conducted both cross-sectional and longitudinal analysis.

**Table 2:** Means, SD, and mean changes by intervention condition.

Condition	Job performance			Work life conflict		
	Time 1 (pre)	Time 2 (post)	Mean change	Time 1 (pre)	Time 2 (post)	Mean change
Positive impact reflection	4.59 (0.45)	4.52_b_ (0.48)	0.07	2.95 (1.01)	2.75_a_ (0.94)	0.20^+^
Negative impact reflection	4.61 (0.44)	4.36_a,b_ (0.47)	0.25*	3.05 (0.91)	3.08_a_ (0.97)	0.03
Control	4.63 (0.46)	4.53_a_ (0.47)	0.10*	2.76 (0.94)	2.91 (1.12)	0.15^+^

SDs are in parentheses. *Mean changes are significant at *p* < 0.05; ^+^mean changes are significant at the *p* < 0.10 level. Means with the same subscript are significant atthe *p* < 0.05 level.

### CROSS SECTIONAL ANALYSES

#### Pre-intervention

Omnibus ANOVAs showed that the three conditions did not differ 1-week prior to the start of the intervention in job performance, *F*(2,216) = 0.122, *p* = 0.89, η^2^ = 0.01, or in work-life conflict, *F*(2,216) = 1.73, *p* = 0.18, η^2^ = 0.02. Results of contrast analyses revealed no significant differences between individual conditions on the pre-intervention measures (see **Table 2**).

#### Post intervention

Omnibus ANOVAs indicated marginally significant differences between conditions 1-week following the intervention in job performance, *F*(2,175) = 2.35, *p* = 0.10, η^2^ = 0.03, and no significant differences in work-life conflict, *F*(2,173) = 1.47, *p* = 0.23, η^2^ = 0.02. Contrast analyses revealed that, following the intervention, professionals in the negative impact reflection condition reported significantly lower levels of job performance than those in the control condition, *t*(109) = -1.95, *p* = 0.05, η^2^ = 0.03, and those in the positive impact reflection condition, *t*(114) = -1.88, *p* = 0.05, η^2^ = 0.03. Moreover, professionals in the positive impact reflection condition showed reported levels of work-life conflict that were significantly lower (albeit marginally) than those in the negative impact reflection condition, *t*(112) = 1.82, *p* = 0.07, η^2^ = 0.03, but not significantly lower than those in the control condition, *t*(122) = 0.84, *p* = 0.40, η^2^ = 0.01. Because the gender differences in attrition described previously could have consequences for the interpretation of results, I conducted supplementary analyses controlling for gender. The results showed that controlling for gender did not alter the significance of the findings.

### LONGITUDINAL ANALYSES

I examined differences across the three conditions over time in job performance and work-life conflict by conducting repeated measures ANOVAs using codes of 1 for negative impact reflection, 2 for control, and 3 for positive impact reflection. In repeated measures models, the statistical test of the intervention effect is the time by condition interaction, which tests whether baseline to post-intervention change in the dependent variable is greater for the intervention condition than for the comparison condition (s). The analyses indicated significant interactions between time and condition on job performance, *F*(2,173) = 4.61, *p* = 0.01, η^2^ = 0.01, and work-life conflict, *F*(2,171) = 3.76, *p* = 0.03, η^2^ = 0.01.

To facilitate interpretation of these effects, I conducted several planned contrast analyses. A repeated measures ANOVA comparing the positive impact reflection condition with the other two conditions showed a significant time by condition interaction on both performance, *F*(1,174) = 4.76, *p* = 0.03, η^2^ = 0.03, and work-life conflict, *F*(1,172) = 7.13, *p* = 0.01, η^2^= 0.04. A repeated measures ANOVA comparing the negative impact reflection condition to the other two conditions revealed a significant time by condition interaction on job performance, *F*(1,174) = 8.43, *p* = 0.01, η^2^ = 0.05, but not work-life conflict, *F*(1,172) = 0.52, *p* = 0.48, η^2^ = 0.03. Paired samples *t*-tests indicated marginally significant decreases in work-life conflict, *t*(63) = 1.63, *p* = 0.10, η^2^ = 0.01, for those in the positive impact reflection condition, but no significant change in job performance, *t*(63) = 0.95, *p* = 0.34, η^2^ = 0.003. Professionals in the negative impact reflection condition showed significant declines in job performance, *t*(50) = 4.48, *p* = 0.001, η^2^ = 0.23, but not work-life conflict, *t*(49) = -1.04, *p* = 0.30, η^2^ = 0.001. Professionals in the control condition demonstrated significant declines in job performance, *t*(59) = 2.35, *p* = 0.02, η^2^ = 0.02, and marginally significant increases in work-life conflict, *t*(59) = -1.90, *p* = 0.07, η^2^ = 0.01.

Again, because of the noted gender differences in attrition from Time 1 to Time 2, I sought to ensure that the observed temporal effects were not confounded by the changes in gender composition. I conducted supplementary analyses controlling for both the main effect of gender and the interaction between gender and condition. The results showed that controlling for gender did not alter the significance of the findings.

**FIGURE 1:**
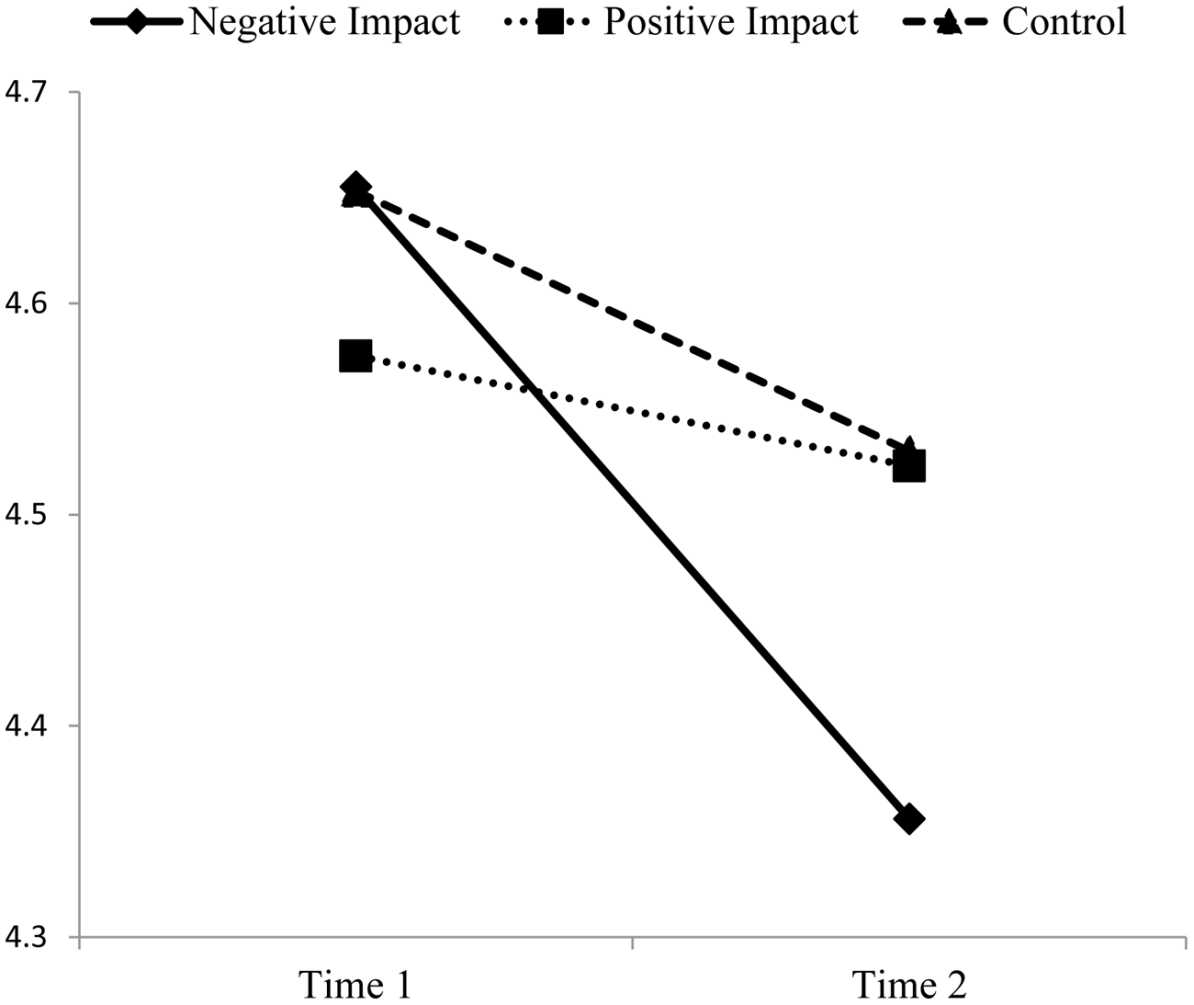
**Job performance pre- and post-intervention**.

**FIGURE 2:**
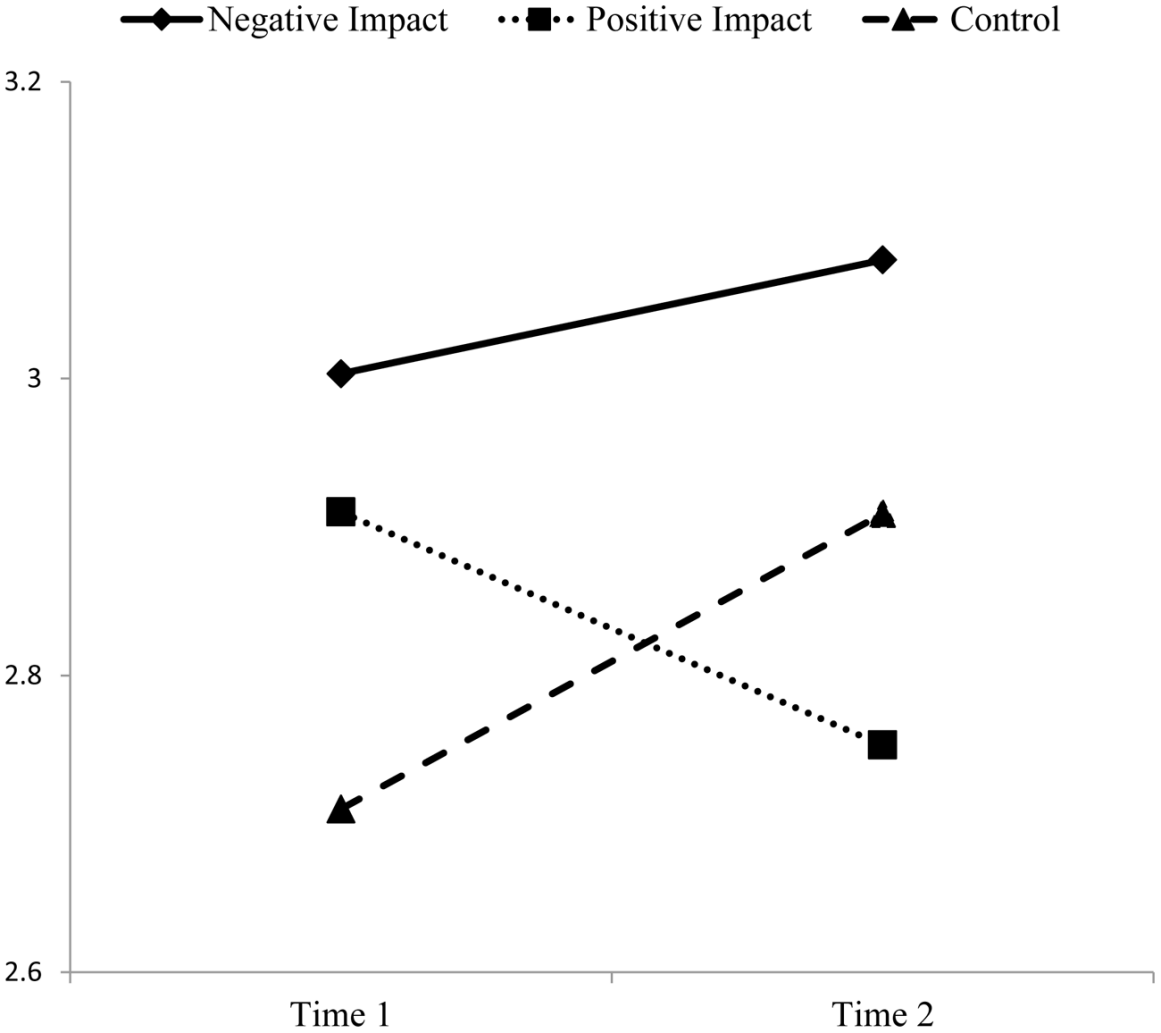
**Work-life conflict pre- and post-intervention**.

Thus, as displayed in **Figures 1** and **2** negative impact reflection had a pronounced negative effect on job performance, but no significant effect on work-life conflict. Positive impact reflection had a significant (positive) effect on work-life conflict compared to the others two conditions, but that change was only marginally significant. Moreover, positive impact reflection had no significant effect on job performance. The direction of effects for the control condition mirrored those of the negative impact reflection condition for both job performance and work-life conflict.

## DISCUSSION

The purpose of this study was to test the idea that reflecting on the positive and negative impact of one’s existing job could affect job performance and work-life conflict over time. Based on prior research, I expected to find that reflecting on positive impact at work would lead to increases in self-rated job performance and decreases in work-life conflict perceptions. Results show that the manipulations worked, and reveal some intervention effects. I found evidence that negative impact reflection had a significant (negative) effect on job performance, one that was more pronounced than the negative effect on job performance seen in the control condition. Positive impact reflection resulted in significant (positive) change to work-life; however, the change from pre-intervention to post intervention was only marginally significant. The direction of relationships in the control condition mirrored the direction of effects in the negative impact reflection condition.

### THEORETICAL IMPLICATIONS

This research has several theoretical implications for the literature on relational job design and task significance (i.e., literature focused on the effect of impact perceptions). The first contribution lies in elaborating the causal effects of regular impact reflection on job performance and work-life conflict. As past research testing the effect of impact interventions has focused primarily on the positive effects of connecting individuals with the beneficiaries of their work (Grant, 2008a), this research tested the effect of another type of intervention – engaging employees in regular reflection about how their existing work benefits others. Interestingly, while previous research has shown that one-time interventions, designed to put employees in touch with the beneficiaries of their work, have a positive effect on job performance (Grant et al., 2007), I found that repeated positive impact reflection had no significant effect on job performance. Moreover, the study results with respect to the effect of positive impact cues on work-life conflict were in line with previous research, but showed a weaker effect than that reported by others (Sonnentag and Grant, 2012).

Although future research is needed to resolve these incongruent findings, two potential explanations for the differences may be the repetition of impact cuing and the self-ratings of job performance. First, in previous field experiments involving positive impact cues, participants engaged in a one-time intervention (Grant et al., 2007; Grant, 2008a). In this study however, participants engaged in repeated reflection over an 8 week period. Previous research on repetition priming suggests that priming can be a transient phenomenon that saturates after a number of repetitions (Hauptmann and Karni, 2002). Thus, a longer intervention period may have caused workers to become desensitized to positive impact cues. Second, while previous research has used objective measures of performance (e.g., Grant et al., 2007), desensitization to positive impact cues may be especially likely with self-report measures, such as those used in this study. This raises the possibility that repeated impact cues may uniquely effect employee evaluations of performance.

A second theoretical contribution of this study lies in elaborating the effects of reflection about both positive and negative impact on others. While cross-sectional research has tested the effect of present perceptions of negative impact (Grant and Campbell, 2007), this study tested whether engaging in regular reflection about one’s negative impact at work was consequential to changes in job performance and work-life conflict. I found that those in the negative impact reflection condition had significant declines in job performance, but no significant increases in work-life conflict during the intervention period. Though I found a similar patterns of changes for those in the negative impact reflection and control conditions, job performance declines were more pronounced in the negative impact reflection condition, suggesting that negative impact reflection may have accelerated a sample tendency toward declining performance.

The pattern of results also suggests that those in the positive impact reflection condition deviated from the sample tendency toward declines in job performance ratings, and increases in employee work-life conflict ratings. These findings, combined with evidence that employee attitudes and performance may naturally fluctuate over time (Fisher and Ashkanasy, 2000; Wright and Bonnet, 2002), raise the interesting possibility that positive impact reflection may produce something akin to a “buffering effect” (Newton and Teo, 2014), such that employees who engage in regular positive impact reflection are somehow buffered from the evaluative judgments that produce regular fluctuations in employee job performance and work-life conflict. Just as cross-sectional studies have shown that positive impact may mitigate the effect of negative impact on employee burnout (Grant and Campbell, 2007) and that identification can buffer employees against workplace stress (Newton and Teo, 2014), positive impact reflection may buffer employees against negative evaluations of their experiences at work and outside of work. Of course, this possibility requires further investigation, but by examining the effect of positive and negative impact reflection together, this study adds additional nuance to the understanding of how positive impact cues may affect employee job performance and work-life conflict.

A third theoretical contribution lies in furthering an understanding of social information processing perspectives on task significance. While previous research has tested the effect of information from beneficiaries (Grant et al., 2007; Grant, 2008a), this research tested the effect of reflecting about beneficiary impact. Combining previous research with the effects of positive impact reflection found in this study, it appears that receiving information directly, or even indirectly, *from* actual beneficiaries about the positive impact of one’s work (e.g., through contact with and stories about beneficiaries) may exert a stronger effect on employee performance than simply reflecting *about* one’s positive impact on others. This may be because information directly from beneficiaries may make one’s impact more salient and provide tangible feedback that one’s actions are having a positive impact, thereby motivating employees to exert additional effort (Grant, 2008a). In contrast, reflection may focus workers on the possibility of positive impact without providing the salient or tangible evidence that such impact actually occurred. With respect to work-life conflict, it appears that positive impact reflection is enough to produce some change in people’s perceptions of the tension between work and home. One explanation may be that simply thinking about one’s positive impact may produce some increases in positive affect which spills over from work to home (Sonnentag and Grant, 2012). However, it appears that thinking about negative impact does not cause a negative spillover effect. This may be because individuals are more likely to rationalize their negative impact to neutralize its effect on psychological well-being (James and LeBreton, 2010). Taken together, the study findings suggest that researchers interested in fostering task significance perceptions through impact interventions need to further examine how and why different forms of information processing with respect to beneficiaries exert their effect on employees.

### LIMITATIONS AND FUTURE RESEARCH

This study has limitations that provide additional avenues for future research. First, while I used a longitudinal field experimental methodology, and sampled professionals from multiple organizational contexts, one limitation is the study’s reliance on self-reported outcomes. While the measurement of changes in job performance and work-life conflict, and the inclusion of a control condition, enhance an ability to make conclusions about causality, future studies should attempt to capture supervisor ratings of the outcomes measured, as well as additional outcomes, to build on the results presented here.

Second, while I was able to engage a group of professionals in impact reflection over an 8-week period, I was not able to test the effect of other impact reflection periods of shorter or longer duration. As noted, this raises the possibility that impact reflection of a shorter, or longer, duration may produce different results. Future studies should explore this possibility. Similarly, while this is the first study to my knowledge to test the effect of impact reflection in a sample of professionals from multiple organizational contexts, my sample was comprised of professionals trained in human resources management and industrial relations. Thus, future research is needed to test the effect of impact reflection in other occupational groups.

Third, it is possible that the higher attrition rate for men could have affected the results. Similarly, the higher attrition rate in the negative impact reflection condition may have impacted the results reported here. I found that those in the negative impact reflection condition were both more likely to leave the study, and to report fewer instances of impact during the weekly reflections. These findings point to at least two possibilities. The first is that individuals may prefer to avoid engaging in repeated negative impact reflection because it is difficult or uncomfortable (Margolis and Molinsky, 2008). The second is that individuals may have a more difficult time recalling instances of negative impact, perhaps because individuals try to neutralize the personal effects of negative thoughts and actions (James and LeBreton, 2010). In either case, selective attrition may have influenced the results.

## CONCLUSION

Researchers and practitioners alike are interested in how to foster employee perceptions of impact through work, and the associated benefits. This paper presented results from a longitudinal field experiment examining the effect of engaging employees in regular positive and negative impact reflection. Negative impact reflection had a strong negative effect on job performance, but no effect on work-life conflict. Positive impact reflection had a weak positive effect on work-life conflict, but no effect on job performance. The pattern of results seen in the no intervention condition mirrored that of the negative impact reflection condition, suggesting a possible buffering effect for positive impact reflection. These results, combined with previous research, suggest opportunities for additional research investigating the effect of both positive and negative impact interventions across different methodologies, time periods, and contexts.

## Conflict of Interest Statement

The author declares that the research was conducted in the absence of any commercial or financial relationships that could be construed as a potential conflict of interest.

## Footnotes

1 For the negative impact reflection condition, the percentage of women at Time 1 and Time 2 was 55.4 and 56.9%, respectively. For the positive impact reflection condition, the percentage of women at Time 1 and Time 2 was 73.4 and 78.3%, respectively. For the control condition, the percentage of women at Time 1 and Time 2 was 65.8 and 67.7%, respectively.
